# Adipose Tissue Macrophages of the Human Fetus

**DOI:** 10.3390/cells13211787

**Published:** 2024-10-28

**Authors:** Ádám Radványi, Katalin Gyurina, Emese Rácz, Ilona Kovács, Gábor Méhes, Tamás Röszer

**Affiliations:** 1 Department of Pediatrics, Faculty of Medicine, University of Debrecen, 4032 Debrecen, Hungary; 2 Department of Pathology, Faculty of Medicine, University of Debrecen, 4032 Debrecen, Hungary

**Keywords:** adipose tissue, macrophages, immunity, metabolism, human development

## Abstract

Prenatal adipose tissue development affects body composition and growth trajectory in early infancy, therefore it is a key determinant of adiposity in childhood. Childhood overweight and obesity increase the probability of being obese as an adult. After birth and in adulthood, adipose tissue macrophages (ATMs) are relevant constituents of the fat depots, and they are necessary for physiological adipose tissue development and fat metabolism. In obesity, however, ATMs may induce chronic inflammation leading to insulin resistance, pancreatic beta cell damage and self-immunity. Despite being relevant regulators of adipose tissue development and functioning, it is unknown whether ATMs are present in the fetal adipose tissue, therefore it is elusive whether they may affect the prenatal establishment of fat depots. Here we studied the distribution of ATMs in the human fetus between gestational weeks 17 and 38 and labeled ATMs in the early postnatal life. We found that CD45^+^/CD14^+^/CD68^+^ ATMs infiltrated the fetal adipose tissue from the 17th week of gestation and remained persistent throughout the second and third trimesters. ATMs were phagocytic in the neonate and expressed interleukin-6, along with other pro-inflammatory gene products. These findings show that ATMs colonize the adipose tissue early in gestation, raising the possibility that intrauterine ATM–adipocyte communication may exist, eventually allowing ATMs to affect prenatal adipose tissue development.

## 1. Introduction

Adipose tissue macrophages (ATMs) are resident myeloid cells of the adipose tissue that constitute approximately 10% of the adipose tissue stroma under physiological conditions [1]. In obesity, hypertrophic adipocytes emit chemotactic and pro-inflammatory signals, which boost monocyte development in the bone marrow, enhance monocyte migration to the fat depots, induce local ATM proliferation and ultimately increase the number of ATMs [2]. Therefore, the prevalence of ATMs may increase up to 50% in severe obesity, and ATMs fuse to form so-called crown-like structures around hypertrophic and eventually dying fat cells [3,4]. These crown-like structures release pro-inflammatory mediators that may trigger chronic inflammation. ATMs in obesity hence induce adipose tissue inflammation, also known as metabolic inflammation, that may cause insulin resistance, pancreatic beta cell damage and self-immunity against certain molecules of the adipocytes, leading to metabolic diseases associated with obesity [5].

ATMs were initially observed in obese adipose tissue, and for this reason obesity-associated roles of ATMs have been well explored [1,3]. However, much less is known about the physiological functions of ATMs in lean adipose tissue. Similarly to other tissue resident macrophages, ATMs may clear apoptotic cells, immune complexes and pathogens in the adipose tissue [6]. Beyond these broad immune surveillance tasks, ATMs may have specific effects that are vital for metabolic health. For instance, they are necessary for postnatal adipocyte development and maintenance of thermogenic competence of the young adipocytes [7]. Ablation of ATMs in newborn mice causes a loss of thermogenic fat cells in the subcutaneous adipose tissue [8]. The underlying mechanism is an ATM-dependent conversion of human milk-derived lipids into mediators that promote thermogenesis [8]. It is also thought that ATMs release pro-resolving mediators—such as interleukin-10 and interleukin-1 receptor antagonist—that prevent adipose tissue inflammation [9,10], and control neurotransmitter levels in the adipose tissue that are necessary for lipolysis and thermogenesis [11,12].

Each organ has its unique combination of macrophages that are descendants of embryonic hematopoietic cells and bone marrow-derived monocytes [13,14]. A fate mapping study shows that the first wave of ATMs originates from embryonic hematopoietic precursors in mouse [15]. Later in life and during obesity progression, these ATMs are replaced by bone marrow-derived monocytes, suggesting that recruited monocytes are the primary contributors to the increased number of ATMs in obesity [2]. ATMs have been detected in human infants, and an increased number of ATMs is associated with childhood obesity [8,16]. However, no study has addressed the question of whether ATMs appear before birth, during the initial phase of adipose tissue differentiation. Unlike in many other mammals—including mice that are widely used animal models of human obesity—the subcutaneous fat depots begin to develop during the second and third trimesters in the human fetus [17]. At birth, adipose tissue serves as a relevant source for energy production, including heat generation [18,19,20], and gradually transforms into an energy reserve and endocrine organ [21,22]. It is unknown whether ATMs are present during the early adipose tissue development, hence any role for ATMs in fetal adipose tissue expansion and adiposity state at birth is elusive to date.

The aim of this study was to identify ATMs during the human intrauterine development. We used immunohistochemistry to detect the expression of macrophage-associated proteins in the developing adipose tissue of human fetuses between gestational weeks 17 and 38. We also used adipose tissue biopsies from human infants and children to isolate ATMs and determine their phagocytosis capacity and expression levels of macrophage-associated proteins, as well as to describe their gene expression profiles, with the use of next-generation sequencing.

## 2. Materials and Methods

### 2.1. Human Samples

**Figure 1:**
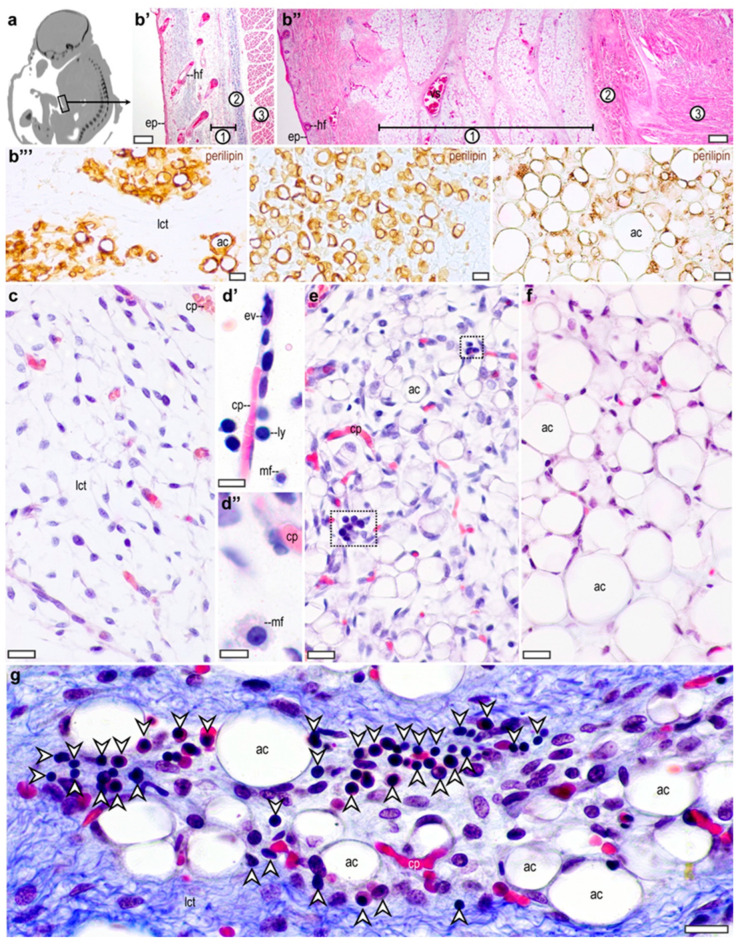
Development of the subcutaneous adipose tissue depot of the abdominal wall in the human fetus. (**a**) Computed tomography image showing sagittal section of a human fetus in the late third trimester. Abdominal wall at the level of the umbilical cord is indicated in frame. Original image, retrieved from a postmortem computed tomography scan performed in our previous study [25]. (**b’**) Cross section of the abdominal wall of the human fetus at the 17th week of gestation. ep: epidermis, hf: hair follicle, 1: subcutaneous connective tissue layer with preadipocytes, 2: collagen fibers, 3: muscular wall of the abdomen. Masson’s trichromic staining. Scale bar: 200 μm. (**b’’**) Cross section of the abdominal wall in the third trimester. ep: epidermis, hf: hair follicle, vs: vesicles, 1: subcutaneous fat layer, 2: collagen fibers, 3: muscular wall of the abdomen. Hematoxylin and eosin staining. Scale bar: 200 μm. (**b’’’**) Perilipin immunostaining of the developing adipose tissue. From left to right: week 17, week 21 and week 38. ac: adipocyte, lct: loose connective tissue. Scale bar 50 μm. (**c**) Subcutaneous connective tissue layer of the abdominal wall from the second trimester (17th week) of gestation. cp: capillary, lct: loose connective tissue. Masson’s trichromic staining. Scale bar: 30 μm. (**d’**) Capillary from the subcutaneous connective tissue at the 20th gestational week. ev: extravasating leukocyte, cp: capillary, ly: lymphocyte, mf: macrophage, scale bar 30 μm. (**d’’**) Macrophage (mf) in the subcutaneous connective tissue at the 20th gestational week. cp: capillary. Scale bar 5 μm. (**e**) Subcutaneous adipose tissue at the 21st week of gestation. ac: adipocyte, cp: capillary. Dotted lines mark regions with immune cell infiltration. Masson’s trichromic staining. Scale bar 30 μm. (**f**) Subcutaneous adipose tissue at the 30th week of gestation. ac: adipocyte, Masson’s trichromic staining. Scale bar 30 μm. (**g**) Infiltrating immune cells in the subcutaneous adipose tissue at gestational week 23. lct: loose connective tissue, ac: adipocyte, arrowheads label immune cells. Scale bar 50 μm.

Fetal adipose tissue samples were collected during autopsy of intrauterine fatalities (Table S1). Miscarriages occur more frequently in male fetuses, and this sampling bias is particularly evident in early pregnancies, where the male-to-female ratio is higher at conception but decreases as pregnancies progress, largely due to the higher rate of miscarriages among male fetuses [23,24]. As a routine procedure, fetal bodies and fetal appendices were fixed in formaldehyde solution and were subject to autopsy. For histology analysis, samples were collected from the abdominal wall and, following an additional fixation in 4% paraformaldehyde, diluted in phosphate buffered saline (PBS) and embedded in paraffin. A postmortem computed tomography (CT) scan was performed on a museum specimen of an in utero male fetus in our previous study [25], and an original image was rendered to illustrate the anatomical site of the fat depot, as shown in Figure 1a. The image was reconstructed using RadiAnt DICOM Viewer 2023.1 (Medixant, Burgdorf, Germany), and to ensure sample anonymity, patient information was removed digitally from the image. Postnatal adipose tissue samples were collected during elective surgery from adipose tissue specimens of infants and children. Samples were obtained during elective surgeries performed between June 2022–September 2023 at the Department of Pediatrics, University of Debrecen. Surgical indications were orchidopexy, umbilical, or abdominal hernia (*n* = 16, males: 11, females: 5). Perinatal surgical indications are more frequent among male newborns due to sex-specific surgical indications (e.g., inguinal hernia and orchidopexy). Selection criteria are described elsewhere [8]. We excluded patients with known diabetes or immune conditions, maternal diabetes, bleeding and hematological disorders, oncological disease and cachexia. Acute infections and patient or maternal PCR-positivity for SARS-CoV-2 were additional exclusion criteria. Adipose tissue specimens were fixed in 4% paraformaldehyde, diluted in PBS and embedded in paraffin.

### 2.2. Histology, Immunohistochemistry and Transmission Electron Microscopy

Paraffin blocks were cut with a microtome to collect 7 μm thick sections. The specimens were stained with hematoxylin and eosin and Masson’s trichrome staining (BioGnost, Zagreb, Croatia). We used polyclonal antibodies raised in mouse or rabbit against human perilipin-1 (1:200, #690156S, Progen, Heidelberg, Germany), CD45 (1:100, #Mob040-01, Diagnostic Biosystems Inc., Pleasanton, CA, USA), CD68 (1 μg/mL, #14-0688-82, eBioscience, San Diego, CA, USA) and CD14 (1:500, #HPA001887 Merck Sigma-Aldrich, St. Louis, MO, USA). Antibody binding was visualized with a horseradish peroxidase-conjugated secondary antibody and staining with Impact DAB (Vector Labs, Burlingame, CA, USA). The specificity of the immunostaining was validated by using fetal thymus and lymph node sections. For transmission electron microscopy (TEM) analysis, we fixed tissue samples in paraformaldehyde–glutaraldehyde and processed as described [8]. Semi-thin sections were stained with methylene blue.

### 2.3. Isolation of ATMs and Flow Cytometry

We used fat specimens collected during elective surgeries, since it was not possible to separate ATMs from adipocytes with enzymatic digestion in prenatal samples, owing to the formalin fixation applied before autopsies. Adipocytes and the stromal vascular fraction cells were isolated by collagenase digestion of the adipose tissue specimens, as described [25]. Both ATMs and adipocytes were used for subsequent flow cytometry analysis. Cells were labeled with mouse monoclonal antibodies against CD68 (1:100, #14-0688-82, eBioscience), CD36 (1:100, #11-0369-41, eBioscience) and IL-6 (1:100, #17-7069-41, eBioscience), or with corresponding isotype control immunoglobulins, and analyzed with a BD LSR II flow cytometer (BD Biosciences, Franklin Lakes, NJ, USA), using FACS Diva v8.0 (BD Biosciences, Franklin Lakes, NJ, USA) and FlowJo10.8.1.(FlowJo LLC, Ashland, OR, USA) software for analysis and data visualization. For assessment of phagocytosis, SVF cells were incubated with FITC-conjugated latex beads (Merck Sigma-Aldrich) for 1 h and phagocytic index was calculated as described [26]. Cells were centrifuged and washed with PBS and analyzed with flow cytometry. Raw data are deposited in FlowRepository, under accession number FR-FCM-Z84Y.

### 2.4. Animals

We collected inguinal adipose tissue specimens from young and adult male C57/BL6 mice as described [27] on postnatal day 6 (P6) and 56 (P56), respectively. We used standard, specific pathogen-free housing conditions under ambient temperature, and ad libitum feeding with standard rodent chow diet. Since epididymal adipose tissue expands after weaning age in mice, it is not suitable to study ATM development. To obtain sufficient ATMs, we pooled fat specimens from 6 mice at P6, and 3 mice at P56. We repeated the ATM isolation protocol at three independent assays. Once ATMs were isolated, total RNA was purified and subsequently analyzed with next-generation sequencing.

### 2.5. Gene Expression Analysis

Postmortem samples were not suitable for RNA extraction, therefore we used fat samples collected during elective surgery for analysis of gene expression. Total RNA was extracted using TRIzol reagent (Merck Sigma-Aldrich), as described [27]. Next-generation sequencing was performed on a BGISEQ-500 platform by BGI Genomics Inc. (Cambridge, MA), as described [8]. We used 3/3 adipose tissue samples from human male infants (mean age 13.6 months) and children (mean age 9 years), respectively. For analysis of mouse ATM gene expression, we used 3/3 ATM samples from P6 and P56 mice. We applied log2 transformed fold change to determine gene expression level differences between groups. Differentially expressed genes (DEGs) were identified using the DEseq2 algorithm [28]. We used the STRING Functional Protein Associations Network to visualize protein-protein interaction networks [29]. The raw NGS data are available in NIH GEO under accession numbers GSE274818 and GSE271341.

### 2.6. Statistical Analysis

Data are represented as mean ± standard error of the mean. We used the GraphPad Prism v5.0 for Windows statistical software to visualize and analyze data (GraphPad Software, San Diego, CA, USA). The unpaired 2-tailed Student’s *t*-test was used to compare groups, while correlation between two variables was determined with Gaussian *p*-value approximation, using a 2-tailed Pearson test, with a 95% confidence interval (*α* = 0.05).

## 3. Results

### 3.1. Adipose Tissue Development in the Human Fetus

We analyzed the subcutaneous adipose tissue in the abdominal wall, at the level of the umbilical–hypogastric area (Figure 1a). The subcutaneous tissue layer was formed by a loose connective tissue in the early stage of the second trimester (Figure 1b’), which expanded into a fat layer in the third trimester (Figure 1b’’). Perilipin expressing preadipocyte clusters were apparent at around the 17th gestational week, increasing in number and cell size throughout the intrauterine life (Figure 1b’’’). Both unilocular and multilocular adipocytes were present in the fetal fat depot (Figure 1b’’’). Within the subcutaneous loose connective tissue (Figure 1c), we found macrophage-like cells, mostly in the vicinity of capillaries during the early second trimester (Figure 1d’,d’’). Immune cells with rounded nuclei were detectable within the preadipocyte clusters from the second trimester onwards (Figure 1e–g).

### 3.2. CD45-Expressing Cells in the Developing Adipose Tissue

First, we aimed to detect all leukocytes in the developing adipose tissue. We selected the leukocyte common antigen CD45, also termed as protein tyrosine phosphatase receptor type C, as a suitable marker to label leukocytes [30]. CD45 is expressed on leukocytes with lymphoid and myeloid origin, and in mouse adipose tissue, ATMs are defined as CD45^+^ cells [31]. CD45 denotes at least four glycoproteins of different molecular weights [32]. We used a monoclonal antibody that recognizes epitopes on all forms of CD45. As a positive control, we used human fetal thymus and lymph node samples (Figure S1).

**Figure 2:**
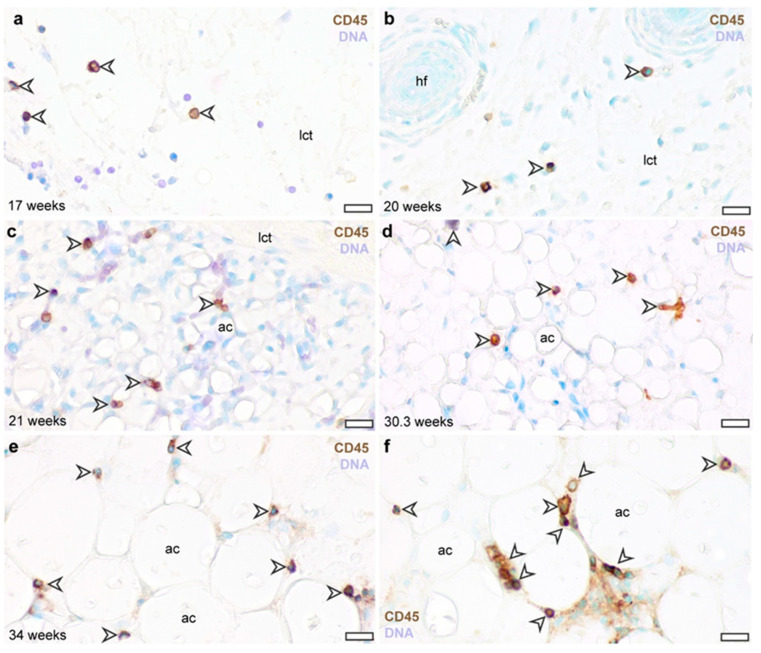
CD45 immunostaining of the abdominal subcutaneous adipose tissue in the human fetus. (**a**) Subcutaneous connective tissue at gestation week 17. (**b**) Subcutaneous connective tissue at gestation week 20. (**c**) Developing subcutaneous adipose tissue depot at gestation week 21. (**d**) Subcutaneous adipose tissue at gestational week 30. (**e**) Subcutaneous adipose tissue at gestational week 34. (**f**) Subcutaneous adipose tissue of a newborn (born at completed gestational week 36). Arrowheads label CD45^+^ cells. lct: loose connective tissue, ac: adipocyte, hf: hair follicle. DNA stained with methyl green. Scale bar 30 μm.

From the 17th week of gestation onwards, CD45-expressing cells were abundant in the developing dermis (Figure 2). These cells were scattered in the loose connective tissue layer beneath the epidermis, often in proximity to hair follicles (Figure 2a,b). Throughout the second trimester, 17.3 ± 3.3% of the stromal cells of the subcutaneous connective tissue layer was positive for CD45. CD45-expressing cells were rounded in shape, with centrally located, rounded and euchromatic nuclei. From the 21st week of gestation, CD45-expressing cells were enriched in the cell clusters formed by developing adipocytes (Figure 2c). The CD45-expressing cells were attached to adipocytes. This morphology and distribution pattern prevailed to the time of transition from the second to the third trimester of gestation (Figure 2d).

In the third trimester, when the adipose tissue volume expansion was apparent, CD45-positive cells appeared adjacent to adipocytes (Figure 2e), and often formed cell clusters (Figure 2f). In this period of adipose tissue development, 52.9 ± 2.4% of adipocytes were associated with at least one CD45-expressing cell.

### 3.3. Distribution of CD68-Expressing Cells in the Developing Adipose Tissue

Human CD68 belongs to the family of lysosomal-associated membrane proteins located in the endosomal and lysosomal membrane and may be shuttled to the cell membrane. CD68 is expressed by human macrophages (Figure S1), and is involved in binding of oxidized low density lipoprotein and phosphatidylserine in apoptotic cell membranes [33].

**Figure 3:**
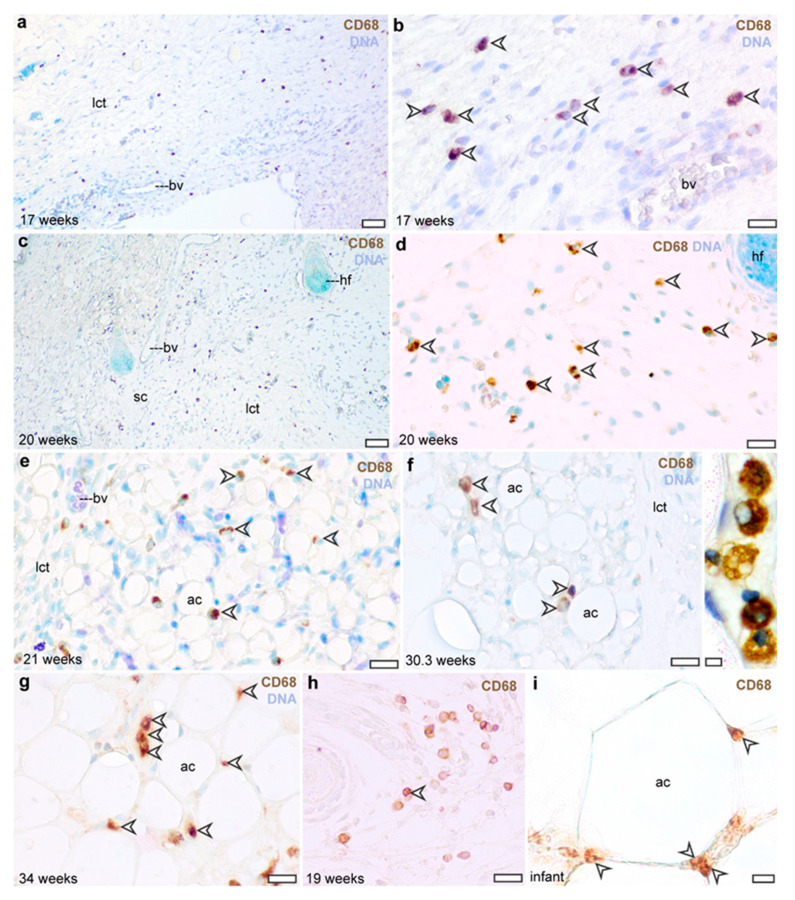
CD68 immunostaining of the abdominal subcutaneous adipose tissue in the human fetus. (**a**,**b**) Subcutaneous connective tissue at gestation week 17. Arrowheads label CD68^+^ cells. Scale bar 50 μm (**a**) and 30 μm (**b**). (**c**,**d**) Subcutaneous connective tissue at gestation week 20. Scale bar 50 μm (**c**) and 30 μm (**d**). (**e**) Developing subcutaneous adipose tissue depot at gestation week 21. Scale bar 30 μm. (**f**) Subcutaneous adipose tissue at gestation week 30. Inset shows a cluster of CD68^+^ cells in vicinity of adipocytes. Scale bar 30 μm and 5 μm in the inlet. (**g**) Subcutaneous adipose tissue at gestational week 34. Scale bar 30 μm. (**h**,**i**) Comparison of the distribution pattern of CD68^+^ cells in the second trimester (**h**) and in the newborn (**i**); scale bar 30 μm. lct: loose connective tissue, ac: adipocyte, hf: hair follicle, sc: subcutis, bv: blood vessel.

Similarly to the distribution pattern of the CD45-expressing cells, CD68-positive cells were detectable from the 17th gestational week in the subcutaneous connective tissue (Figure 3a,b), making 17 ± 3.7% of the stromal cells. These CD68^+^ cells were detectable throughout the second trimester (Figure 3c,d). In the third trimester, the CD68-expressing cells were associated with adipocytes, and this distribution pattern remained persistent in the early postnatal life (Figure 3e–g). At this stage, 47 ± 26% of adipocytes were connected to at least one CD68^+^ cell. Coherently with the association of CD68 with endosomes and lysosomes, CD68 immunostaining appeared in intracellular vesicle-like structures, yielding a granular staining pattern of the cytoplasm (Figure 3f). In summary, during the second trimester, CD68 immunostaining appeared in rounded cells that were scattered in the loose connective tissue beneath the skin (Figure 3h), and in the third trimester CD68^+^ cells were attached to adipocytes (Figure 3i), similarly to the distribution pattern of CD45-expressing cells.

### 3.4. Distribution of CD14-Expressing Cells in the Developing Adipose Tissue

Human macrophages also express myeloid cell-specific leucine-rich glycoprotein CD14, which is a cell surface-associated and secreted protein (Figure S1) involved in pathogen recognition and apoptotic cell clearance [34]. Some of the tissue-resident macrophages of the human dermis express CD14. These macrophages are descendants of blood-derived monocytes with a half-life shorter than 6 days [35].

**Figure 4:**
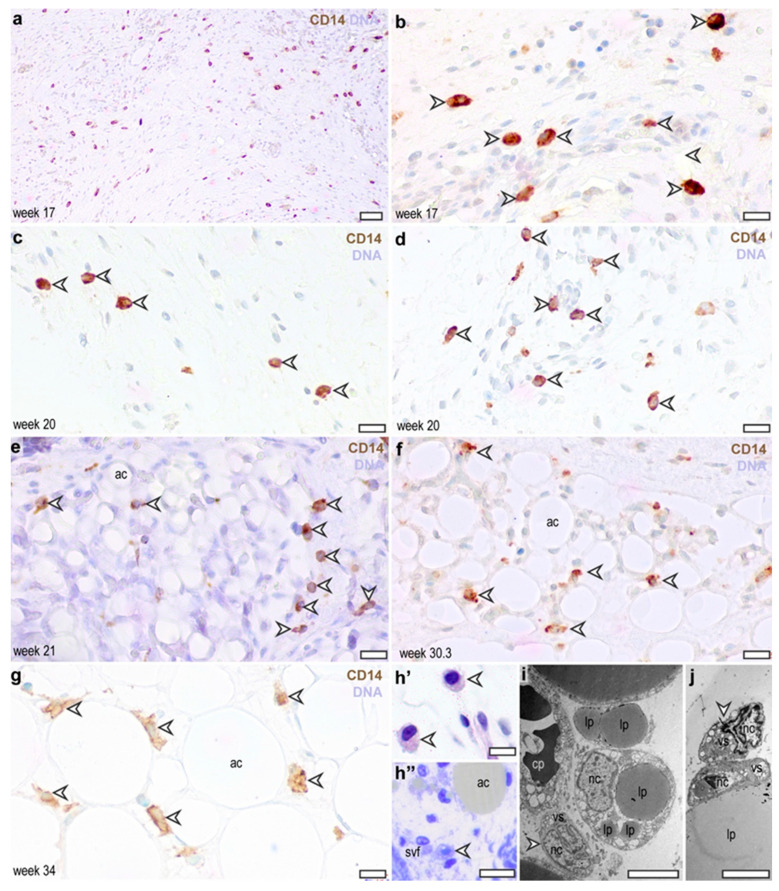
Distribution of CD14-expressing cells in the abdominal subcutaneous adipose tissue in the human fetus. (**a**,**b**) Subcutaneous connective tissue at gestation week 17. Scale bar 50 μm (**a**) and 30 μm (**b**), arrowheads label CD14^+^ cells. (**c**,**d**) Subcutaneous connective tissue at gestation week 20. Arrowheads label CD14^+^ cells. Scale bar 30 μm. (**e**) Developing subcutaneous adipose tissue depot at gestation week 21. ac: adipocyte, arrowheads label CD14^+^ cells. Scale bar 30 μm. (**f**) Subcutaneous adipose tissue at gestation week 30. ac: adipocyte, arrowheads label CD14^+^ cells. Scale bar 30 μm. (**g**) Subcutaneous adipose tissue at gestation week 34. ac: adipocyte, arrowheads label CD14^+^ cells. Scale bar 30 μm. (**h’**,**h”**) Adipose tissue macrophages in the second trimester (**h’**) and in the third trimester (**h”**), methylene blue stained semi-thin sections. Arrowheads label macrophage-like cells. ac: adipocyte, svf: stromal vascular fraction. Scale bar 30 μm. (**i**) Transmission electron microscopy of the subcutaneous adipose tissue, corresponding to the semi-thin sections shown in panels (**h’,h”**), at gestation week 38. A cluster of adipocytes, a capillary (cp) and a macrophage-like cell (arrowhead). lp: lipid droplets in adipocytes, cp: capillary, nc: nucleus, vs: vesicles, scale bar 5 μm. (**j**) Macrophage-like cells attached to adipocytes in the newborn adipose tissue. Scale bar 5 μm.

In the second trimester, CD14-expressing cells were scattered in the connective tissue of the dermis, resembling the distribution patterns of CD45^+^ and CD68^+^ cells (Figure 4a,b). The CD14^+^ cells remained persistent in the connective tissue throughout the second trimester, making 18.6 ± 2.4% of stromal cells (Figure 4c,d). Similar to the CD45^+^ and CD68^+^ cells, CD14+ cells were adjacent to adipocytes in the third trimester and 28.8 ± 0.5% of adipocytes were associated with at least one CD14^+^ cell (Figure 4e–g). Transmission electron microscopy analysis of the adipose tissue suggested the presence of monocyte/macrophage-like cells in the fetal adipose tissue (Figure 4h’–j).

### 3.5. Macrophages of the Newborn Adipose Tissue Express Pro-Inflammatory Genes

**Figure 5:**
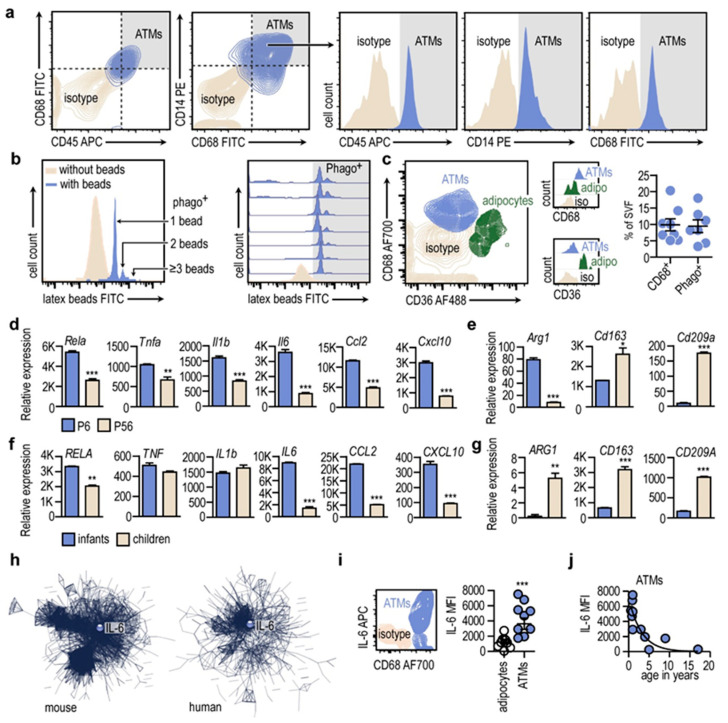
Expression of IL-6 in infant ATMs (**a**) FACS analysis of ATMs in infant adipose tissue. Labeling for CD45, CD68 and CD14. (**b**) Phagocytosis activity of ATMs was assessed with flow cytometry. ATMs engulfed FITC-conjugated latex beads, and fluorescence intensity was proportional to number of ingested beads. Histogram showing fluorescence intensity of ATMs following engulfing latex beads. Phagocytosing cells (Phago^+^) accumulated latex beads, shown by their intensive FITC fluorescence. Each histogram represents one donor patient. (**c**) Expression of CD68 and CD36 in cells of the infant adipose tissue (age range 0.3–0.83 year). Percentage of CD68^+^ and phagocytosing (Phago^+^) cells in the infant adipose tissue. (**d**) Relative mRNA expression levels of M1 macrophage activation genes in mouse ATMs, isolated on postnatal day 6 (P6) and on postnatal day 56 (P56). ** *p* < 0.01, *** *p* < 0.001, 2-tailed unpaired Student’s *t*-test. (**e**) Relative mRNA expression levels of M2 macrophage activation genes in mouse ATMs at P6 and P56. * *p* < 0.05, *** *p* < 0.001, 2-tailed unpaired Student’s *t*-test. (**f**) Relative mRNA expression levels of M1 macrophage activation genes in adipose tissue of human infants and children. ** *p* < 0.01, *** *p* < 0.001, 2-tailed unpaired Student’s *t*-test. (**g**) Relative mRNA expression levels of M2 macrophage activation genes in adipose tissue of human infants and children. ** *p* < 0.01, *** *p* < 0.001, 2-tailed unpaired Student’s *t*-test. (**h**) Interactome map of gene products overrepresented in mouse ATMs on P6 (Left) and in adipose tissue of human infants (Right). (**i**) Expression of IL-6 in ATMs of human infants. Comparison of IL-6 expression (mean fluorescence intensity, MFI) in adipocytes and ATMs. Adipocytes were defined as CD36^+^ cells, ATMs as CD68^+^ cells. Each data point represents one patient. *** *p* < 0.001, 2-tailed unpaired Student’s *t*-test. (**j**) Levels of IL-6 in ATMs.

We continued our analysis with perinatal fat biopsies, obtained during elective surgeries. After birth, CD45-expressing cells were abundant in the stromal vascular fraction of the adipose tissue (Figure 5a). Majority of these cells were expressing macrophage markers CD68 and CD14 (Figure 5a). Using fat specimens from live donors allowed us to measure phagocytic activity of the ATMs. We used fluorescent isothiocyanate (FITC)-conjugated latex beads to measure phagocytosis capacity of the isolated stromal vascular cells (Figure 5b). Phagocytic index was 2.75 ± 0.16, meaning that each ATM ingested at least two to three latex beads during the incubation period (Figure 5b). We found that latex beads were taken up by a similar efficiency in all samples tested, and that the number of phagocytosing cells was similar to the amount of CD68-expressing cells within the stromal vascular fraction (Figure 5b). This suggests that the two populations were identical, and the CD68-expressing cells represented a phagocytosing ATM population. In mice—unlike in humans—the subcutaneous adipose tissue expands in the early postnatal life. At around the 6th postnatal day, the adipose tissue is rich in ATMs, and they are necessary to maintain the thermogenic activity of the adipocytes [8]. We isolated ATMs from young mice at postnatal day 6 (P6) and adult mice at postnatal day 56 (P56), and determined their transcriptional landscape using next-generation RNA sequencing (NGS).

Understanding of macrophage functions is influenced by the M1/M2 macrophage polarization model [36], and M1 traits of ATMs have been linked to obesity and the development of metabolic meta-inflammation [1]. However, inflammatory cytokines have been shown to play a role in physiological adipose tissue development [8,37]. We asked whether P6 ATMs show M1 or M2 traits. We found that P6 ATMs expressed higher levels of M1 marker genes than their P56 counterparts. These mRNA species included *Tnfa*, encoding tumor necrosis factor alpha (TNFα); *Il6*, encoding interleukin-6 (IL-6); *Il1b*, encoding interleukin 1 beta (IL-1β); *Ccl2*, encoding monocyte chemotactic protein (MCP-1); *Cxcl10*, encoding C-X-C motif chemokine ligand 10 (CXCL-10); and *Rela*, encoding nuclear factor kappa B (NFκB) subunit (Figure 5d). Expression of these genes is a hallmark of M1 macrophage activation, and M1 ATMs are associated with the metabolic deterioration caused by obese adipose tissue.

So-called M2, or pro-resolving macrophage activation is hallmarked with the expression of *Arg1*, encoding arginase 1; *Cd163*, encoding CD163 haptoglobin-hemoglobin receptor; and *Cd209a*, encoding CD209 or dendritic cell-specific intercellular adhesion molecule-3-grabbing non-integrin. *Arg1* mRNA level was higher in P6 than in P56 ATMs; however, *Cd163* and *Cd209a* transcript levels were much lower in P6 ATMs than in their P56 counterparts (Figure 5e).

In humans, adipose tissue volume reaches an infancy peak before the first year of age, which is followed by a reduction in fat mass. A surge in fat development appears at the physiological adiposity rebound at 5–6 years of age [38,39]. As a next step, we collected fat specimens from infants before they reached infancy peak of adiposity, and from children passing their adiposity rebound age. We compared the mRNA expression profiles of these samples using NGS.

In infants, the adipose tissue expressed more *RELA*, *IL6*, *CCL2* and *CXCL10*, and less *ARG1*, *CD163* and *CD209A* than the adipose tissue of children (Figure 5f,g). This gene expression difference resembled the situation seen in the postnatal development of mouse ATMs. Indeed, in both young mice and human infants there was a prominent expression of a gene network that contained pro-inflammatory genes and had IL-6 as a central hub within the network (Figure 5h).

While NGS analysis was not feasible to perform on ATMs isolated from fat biopsies, we processed isolated ATMs for flow cytometry. This analysis indicated that IL-6 expression was characteristic to ATMs in the human infant adipose tissue (Figure 5i). Coherently with the NGS data obtained from adipose tissue specimens, IL-6 expression peaked immediately after birth in ATMs (Figure 5j). In summary, human ATMs expressed IL-6 and various M1 macrophage-associated genes after birth.

## 4. Discussion

This study is to our knowledge the first which describes ATMs in the human fetus. Our results show that CD45^+^ leukocytes infiltrate the fetal adipose tissue from the 17th week of gestation and remain persistent throughout the second and third trimesters. CD68^+^ and CD14^+^ macrophages follow a similar pattern and are present in the developing adipose tissue during the second trimester and are closely associated with adipocytes in the third trimester. These findings show that immune cells, particularly ATMs, infiltrate developing adipose tissue early in gestation.

The prenatal development of the immune system is a meticulously orchestrated process, with different immune cells emerging at specific times during gestation [40]. Hematopoietic stem cells (HSCs) are the progenitors of all blood cells and appear in the yolk sac around weeks 3 to 4 of gestation, and some of the tissue resident macrophages are descendants of these HSCs [41,42]. The first definitive HSCs arise from the aorta–gonad–mesonephros region, through a process known as endothelial-to-hematopoietic transition [43]. By week 6, the liver, and later the spleen, emerge as the primary sites of blood cell formation. HSCs express CD34 and CD45. CD34 is expressed on HSCs and progenitors throughout ontogeny, from the early stages of fetal development to the establishment of the adult bone marrow [44]. CD45 is a general marker for leukocytes. Monocytes first appear in the fetal blood between gestational weeks 10 and 12. They typically express CD14 and CD16 markers, while CD68 is often used to identify macrophages derived from the circulating monocytes [45]. In our previous studies, we used ionized calcium-binding adapter molecule 1 (Iba1) and CD163 to label ATMs [8]. While Iba1 is a robust marker for microglia, it is less specific for macrophages in peripheral tissues [46]. CD163 is predominantly expressed on specific macrophage subsets [10]. CD14 and CD68 show more consistent expression across different macrophage populations. In context of human fetal development, macrophages at the maternal-fetal interface are the most studied, and coherently they express CD14 and CD68 [46,47].

Development of the adipose tissue-associated immune cell populations is yet unexplored, and we know little about the establishment of tissue resident macrophage pools in fetal development. The lungs, the adrenal glands, the pancreas, the thyroids, the ovaries, the testes, the liver, the kidneys, the thymus and the skin contain macrophage-like cells during the 13–19th gestational weeks [48]. In addition to placental macrophages, the hepatic macrophage populations are the most studied fetal macrophages, due to their role in the regulation of fetal liver hematopoiesis [49]. Adipose tissue-associated macrophage development, however, appears to be a blind spot in the literature. Human fetal skin contains CD45^+^ and CD68^+^ cells in the second trimester [50], which may be descendants of yolk sac progenitors [51]. It is plausible that, parallel with the macrophage colonization of the skin, the developing subcutaneous fat depot also receives monocytes from the circulation, which may differentiate into ATMs.

Human adipocytes are established before birth, and fat depots begin to expand further in the postnatal life [38,52,53,54]. In contrast, only the interscapular brown adipose tissue is already well developed at birth in mouse. The subcutaneous fat depots are hardly detectable at birth, and they begin to expand until the weaning age [55]. Visceral—epidydimal and omental—fat depots develop later, at around weaning age [56]. In a human newborn, the most relevant fat depots are located subcutaneously and are well-developed at birth [17,18,19,20]. In the early postnatal life, the subcutaneous fat depot contains thermogenic fat cells, supporting the stability of the core body temperature, and providing free fatty acids for energy demanding organs which rely on fatty acid oxidation to gain energy [18,20,25].

It is today accepted in the field that early events of the adipose tissue differentiation and functioning have late-acting effects and determine obesity status in adulthood. For instance, an excessive adipose tissue development in infancy may increase the rebound of adipose tissue expansion in childhood [38,39], which increases the probability of developing overweight or obesity [57,58,59]. ATMs communicate with other immune cells in the adipose tissue and emit signals—cytokines, micro-RNA cargo in extracellular vesicles—which inhibit insulin response and stimulate bone marrow myelopoiesis, leading to a meta-inflammation [9] and insulin resistance [60,61]. Metabolic effects of ATMs are well-explored and understood in the obese adipose tissue, and the prevailing view is that ATMs are metabolically harmful due to their pro-inflammatory nature. This view is, however, challenged by observations showing that some micro-RNA species secreted by ATMs increase insulin sensitivity [60] and promote adipose tissue thermogenesis [62]. Moreover, pro-inflammatory cytokines, such as IL-6 and interferons (IFNs), are necessary for early preadipocyte differentiation and protect from metabolic meta-inflammation [8,27,63,64].

After birth, the mammalian fetus enters a potentially infectious environment from the sterile and immune-surveilled womb. The immune system of the newborn infant hence must protect the body from infections, but also allow the development of the normal microbiome and support tissue differentiation without evoking a potentially damaging inflammation. This duality of functions is unique for the newborn and is seen in the immune gene expression landscape of ATMs in young mice: M1 genes, which are necessary for pathogen elimination, are expressed more prominently than in adults, suggesting a strong immune competence of these macrophages. In turn, M2 markers, which would allow immune evasion of pathogens, are much less expressed in ATMs of young mice. Increased M1 gene expression along with the lack of relevant M2 gene expression suggests a pathogen-eliminating phenotype of the ATMs in newborn mice.

Initiation of labor and parturition is characterized by a surge in inflammatory cytokine synthesis triggering childbirth [65]. Coherently, the adipose tissue of human infants expressed higher levels of pro-inflammatory genes, such as *TNFA*, *IL6*, *IL1B*, *CCL2*, *CXCL10* and *RELA*, and had a suppressed expression of pro-resolving genes, such as *ARG1*, *CD163* and *CD209A*, relative to the adipose tissue of children. Moreover, ATMs in newborn adipose tissue expressed IL-6, and IL-6 was a central hub in the pro-inflammatory gene network expressed by ATMs of young mice and by the adipose tissue of human infants. IL-6 is necessary for the maintenance of the thermogenic potential of the young adipose tissue [8,65], making it plausible that an IL-6-expressing ATM population appears at birth and serves the metabolic demands of heat production in the early postnatal life [7].

## 5. Conclusions

In summary, we show that ATMs are present during the initial phase of adipocyte development in the human fetus, and that ATMs remain persistent during the entire course of adipose tissue differentiation. This makes it possible that fetal ATMs may affect early events of adipose tissue development. Adipose tissue expansion in the first year of life determines the onset of adiposity rebound in early childhood, and an increased adiposity in the early postnatal life may increase the likelihood of overweight and obesity later in life [58,66,67]. The prevalence of childhood obesity makes it key to explore the mechanisms that are responsible for an increased rate of adipose tissue expansion in early postnatal life. Prenatal adipose tissue development affects body composition and growth trajectory in early infancy, therefore it is a key determinant of the adiposity in childhood [57,68,69].

Gestational age, maternal glucose control and obesity determine adiposity in the offspring, and maternal obesity may also induce a pro-inflammatory macrophage activation in the fetus [47,70,71]. In the sensitive period of intrauterine adipose tissue development, various epigenetic factors may increase obesity risk in the offspring, albeit clinical data are limited to date to link maternal obesity status and DNA methylation of genes associated with metabolic development in the newborn [72,73,74]. Moreover, breastfeeding transmits immune regulators that shape macrophage functioning after birth [8,75,76]. It is plausible that a set of maternal cues—e.g., maternal diet, obesity status, composition of milk—may determine ATM activation and metabolic functions before and immediately after birth.

In adult fat depots, ATMs determine adipocyte differentiation potential, fat metabolism and endocrine functioning of the adipose tissue. The present findings on the existence of ATMs in prenatal life raise the possibility that intrauterine ATM–adipocyte communication may exist, which expands the time frame in which ATMs have the possibility to determine adipose tissue development.

## Data Availability

The NGS dataset is available in NIH GEO, under accession numbers GSE274818 and GSE271341. The raw data of FACS analyses are deposited in FlowRepository, under accession number FR-FCM-Z84Y.

## Supplementary Materials

The following supporting information can be downloaded at: https://www.mdpi.com/article/10.3390/cells13211787/s1, Figure S1: Macrophage labeling in fetal thymus and lymph node; Table S1: Gender, gestational age and cause of fatalities.

## References

[1] Boutens L., Stienstra R. (2016). Adipose tissue macrophages: Going off track during obesity. Diabetologia.

[2] Chen Q., Ruedl C. (2020). Obesity retunes turnover kinetics of tissue-resident macrophages in fat. J. Leukoc. Biol..

[3] Weisberg S.P., McCann D., Desai M., Rosenbaum M., Leibel R.L., Ferrante A.W. (2003). Obesity is associated with macrophage accumulation in adipose tissue. J. Clin. Investig..

[4] Hausberger F.X. (1966). Pathological changes in adipose tissue of obese mice. Anat. Rec..

[5] Hildebrandt X., Ibrahim M., Peltzer N. (2023). Cell death and inflammation during obesity: “Know my methods, WAT(son)”. Cell Death Differ..

[6] Rosen E.D., Spiegelman B.M. (2014). What we talk about when we talk about fat. Cell.

[7] Röszer T. (2022). Metabolic impact of adipose tissue macrophages in the early postnatal life. J. Leukoc. Biol..

[8] Yu H., Dilbaz S., Coßmann J., Hoang A.C., Diedrich V., Herwig A., Harauma A., Hoshi Y., Moriguchi T., Landgraf K. (2019). Breast milk alkylglycerols sustain beige adipocytes through adipose tissue macrophages. J. Clin. Investig..

[9] Röszer T. (2020). M2 Macrophages in the Metabolic Organs and in the Neuroendocrine System. The M2 Macrophage.

[10] Zeyda M., Farmer D., Todoric J., Aszmann O., Speiser M., Gyori G., Zlabinger G.J., Stulnig T.M. (2007). Human adipose tissue macrophages are of an anti-inflammatory phenotype but capable of excessive pro-inflammatory mediator production. Int. J. Obes..

[11] Camell C.D., Sander J., Spadaro O., Lee A., Nguyen K.Y., Wing A., Goldberg E.L., Youm Y.H., Brown C.W., Elsworth J. (2017). Inflammasome-driven catecholamine catabolism in macrophages blunts lipolysis during ageing. Nature.

[12] Pirzgalska R.M., Seixas E., Seidman J.S., Link V.M., Sánchez N.M., Mahú I., Mendes R., Gres V., Kubasova N., Morris I. (2017). Sympathetic neuron–associated macrophages contribute to obesity by importing and metabolizing norepinephrine. Nat. Med..

[13] Davies L.C., Jenkins S.J., Allen J.E., Taylor P.R. (2013). Tissue-resident macrophages. Nat. Immunol..

[14] Hashimoto D., Chow A., Noizat C., Teo P., Beasley M.B., Leboeuf M., Becker C.D., See P., Price J., Lucas D. (2013). Tissue resident macrophages self-maintain locally throughout adult life with minimal contribution from circulating monocytes. Immunity.

[15] Waqas S.F.H., Noble A., Hoang A., Ampem G., Popp M., Strauß S., Guille M., Röszer T. (2017). Adipose tissue macrophages develop from bone marrow-independent progenitors in *Xenopus laevis* and mouse. J. Leukoc. Biol..

[16] Landgraf K., Rockstroh D., Wagner I.V., Weise S., Tauscher R., Schwartze J.T., Löffler D., Bühligen U., Wojan M., Till H. (2015). Evidence of early alterations in adipose tissue biology and function and its association with obesity-related inflammation and insulin resistance in children. Diabetes.

[17] Poissonnet C.M., Burdi A.R., Garn S.M. (1984). The chronology of adipose tissue appearance and distribution in the human fetus. Early Hum. Dev..

[18] Herrera E., Amusquivar E. (2000). Lipid metabolism in the fetus and the newborn. Diabetes Metab. Res. Rev..

[19] Whyte R.K., Bayley H.S., Draper H.H. (1990). Energy Metabolism of the Newborn Infant. Advances in Nutritional Research.

[20] Persson B. (1974). Carbohydrate and Lipid Metabolism in the Newborn Infant. Acta Anaesthesiol. Scand..

[21] Symonds M.E., Pope M., Sharkey D., Budge H. (2012). Adipose tissue and fetal programming. Diabetologia.

[22] Galic S., Oakhill J.S., Steinberg G.R. (2010). Adipose tissue as an endocrine organ. Mol. Cell. Endocrinol..

[23] Orzack S.H., Stubblefield J.W., Akmaev V.R., Colls P., Munné S., Scholl T., Steinsaltz D., Zuckerman J.E. (2015). The human sex ratio from conception to birth. Proc. Natl. Acad. Sci. USA.

[24] Boklage C.E. (2005). The epigenetic environment: Secondary sex ratio depends on differential survival in embryogenesis. Hum. Reprod..

[25] Gyurina K., Yarmak M., Sasi-Szabó L., Molnár S., Méhes G., Röszer T. (2023). Loss of Uncoupling Protein 1 Expression in the Subcutaneous Adipose Tissue Predicts Childhood Obesity. Int. J. Mol. Sci..

[26] Santulli-Marotto S., Gervais A., Fisher J., Strake B., Ogden C.A., Riveley C., Giles-Komar J. (2015). Discovering Molecules That Regulate Efferocytosis Using Primary Human Macrophages and High Content Imaging. PLoS ONE.

[27] Hoang A.C., Sasi-Szabó L., Pál T., Szabó T., Diedrich V., Herwig A., Landgraf K., Körner A., Röszer T. (2022). Mitochondrial RNA stimulates beige adipocyte development in young mice. Nat. Metab..

[28] Love M.I., Huber W., Anders S. (2014). Moderated estimation of fold change and dispersion for RNA-seq data with DESeq2. Genome Biol..

[29] Szklarczyk D., Gable A.L., Lyon D., Junge A., Wyder S., Huerta-Cepas J., Simonovic M., Doncheva N.T., Morris J.H., Bork P. (2019). STRING v11: Protein-protein association networks with increased coverage, supporting functional discovery in genome-wide experimental datasets. Nucleic Acids Res..

[30] Dalchau R., Fabre J.W. (1981). Identification with a monoclonal antibody of a predominantly B lymphocyte-specific determinant of the human leukocyte common antigen. Evidence for structural and possible functional diversity of the human leukocyte common molecule. J. Exp. Med..

[31] Cho K.W., Morris D.L., Lumeng C.N. (2014). Flow cytometry analyses of adipose tissue macrophages. Methods Enzym..

[32] Terry L.A., Brown M.H., Beverley P.C. (1988). The monoclonal antibody, UCHL1, recognizes a 180,000 MW component of the human leucocyte-common antigen, CD45. Immunology.

[33] Chistiakov D.A., Bobryshev Y.V., Orekhov A.N. (2016). Macrophage-mediated cholesterol handling in atherosclerosis. J. Cell. Mol. Med..

[34] Devitt A., Pierce S., Oldreive C., Shingler W.H., Gregory C.D. (2003). CD14-dependent clearance of apoptotic cells by human macrophages: The role of phosphatidylserine. Cell Death Differ..

[35] McGovern N., Schlitzer A., Gunawan M., Jardine L., Shin A., Poyner E., Green K., Dickinson R., Wang X.-N., Low D. (2014). Human Dermal CD14^+^ Cells Are a Transient Population of Monocyte-Derived Macrophages. Immunity.

[36] Röszer T. (2015). Understanding the Mysterious M2 Macrophage through Activation Markers and Effector Mechanisms. Mediat. Inflamm..

[37] Sun K., Gao Z., Kolonin M.G. (2018). Transient inflammatory signaling promotes beige adipogenesis. Sci. Signal..

[38] Rolland-Cachera M.F., Deheeger M., Bellisle F., Sempé M., Guilloud-Bataille M., Patois E. (1984). Adiposity rebound in children: A simple indicator for predicting obesity. Am. J. Clin. Nutr..

[39] Dietz W.H. (1994). Critical periods in childhood for the development of obesity. Am. J. Clin. Nutr..

[40] Mor G., Cardenas I. (2010). The Immune System in Pregnancy: A Unique Complexity. Am. J. Reprod. Immunol..

[41] Perdiguero E.G., Klapproth K., Schulz C., Busch K., Azzoni E., Crozet L., Garner H., Trouillet C., de Bruijn M.F., Geissmann F. (2014). Tissue-resident macrophages originate from yolk-sac-derived erythro-myeloid progenitors. Nature.

[42] Lichanska A.M., Hume D.A. (2000). Origins and functions of phagocytes in the embryo. Exp. Hematol..

[43] Ottersbach K. (2019). Endothelial-to-haematopoietic transition: An update on the process of making blood. Biochem. Soc. Trans..

[44] Sidney L.E., Branch M.J., Dunphy S.E., Dua H.S., Hopkinson A. (2014). Concise review: Evidence for CD34 as a common marker for diverse progenitors. Stem Cells.

[45] Hoeffel G., Ginhoux F. (2018). Fetal monocytes and the origins of tissue-resident macrophages. Cell. Immunol..

[46] Jiang X., Du M.R., Li M., Wang H. (2018). Three macrophage subsets are identified in the uterus during early human pregnancy. Cell. Mol. Immunol..

[47] Challier J.C., Basu S., Bintein T., Minium J., Hotmire K., Catalano P.M., Hauguel-de Mouzon S. (2008). Obesity in pregnancy stimulates macrophage accumulation and inflammation in the placenta. Placenta.

[48] Oliver A.M. (1990). Macrophage heterogeneity in human fetal tissue. Fetal macrophages. Clin. Exp. Immunol..

[49] Kayvanjoo A.H., Splichalova I., Bejarano D.A., Huang H. (2024). Fetal liver macrophages contribute to the hematopoietic stem cell niche by controlling granulopoiesis. eLife.

[50] Walraven M., Talhout W., Beelen R.H., van Egmond M., Ulrich M.M. (2016). Healthy human second-trimester fetal skin is deficient in leukocytes and associated homing chemokines. Wound Repair Regen..

[51] Hoeffel G., Wang Y., Greter M., See P., Teo P., Malleret B., Leboeuf M., Low D., Oller G., Almeida F. (2012). Adult Langerhans cells derive predominantly from embryonic fetal liver monocytes with a minor contribution of yolk sac-derived macrophages. J. Exp. Med..

[52] Novak M., Monkus E. (1972). Metabolism of Subcutaneous Adipose Tissue in the Immediate Postnatal Period of Human Newborns. 1. Developmental Changes in Lipolysis and Glycogen Content. Pediatr. Res..

[53] Cunnane S.C., Crawford M.A. (2003). Survival of the fattest: Fat babies were the key to evolution of the large human brain. Comp. Biochem. Physiol. Part A Mol. Integr. Physiol..

[54] Knittle J.L., Timmers K., Ginsberg-Fellner F., Brown R.E., Katz D.P. (1979). The growth of adipose tissue in children and adolescents. Cross-sectional and longitudinal studies of adipose cell number and size. J. Clin. Investig..

[55] Hoang A.C., Yu H., Röszer T. (2021). Transcriptional Landscaping Identifies a Beige Adipocyte Depot in the Newborn Mouse. Cells.

[56] Tsukada A., Okamatsu-Ogura Y., Futagawa E., Habu Y., Takahashi N., Kato-Suzuki M., Kato Y., Ishizuka S., Sonoyama K., Kimura K. (2023). White adipose tissue undergoes browning during preweaning period in association with microbiota formation in mice. iScience.

[57] Yin J., Quinn S., Dwyer T., Ponsonby A.L., Jones G. (2012). Maternal diet, breastfeeding and adolescent body composition: A 16-year prospective study. Eur. J. Clin. Nutr..

[58] Wen X., Kleinman K., Gillman M.W., Rifas-Shiman S.L., Taveras E.M. (2012). Childhood body mass index trajectories: Modeling, characterizing, pairwise correlations and socio-demographic predictors of trajectory characteristics. BMC Med. Res. Methodol..

[59] Geserick M., Vogel M., Gausche R., Lipek T., Spielau U., Keller E., Pfaffle R., Kiess W., Körner A. (2018). Acceleration of BMI in early childhood and risk of sustained obesity. N. Engl. J. Med..

[60] Ying W., Riopel M., Bandyopadhyay G., Dong Y., Birmingham A., Seo J.B., Ofrecio J.M., Wollam J., Hernandez-Carretero A., Fu W. (2017). Adipose Tissue Macrophage-Derived Exosomal miRNAs Can Modulate In Vivo and In Vitro Insulin Sensitivity. Cell.

[61] Patra D., Ramprasad P., Sharma S., Dey U., Kumar V., Singh S., Dasgupta S., Kumar A., Tikoo K., Pal D. (2024). Adipose tissue macrophage-derived microRNA-210-3p disrupts systemic insulin sensitivity by silencing GLUT4 in obesity. J. Biol. Chem..

[62] Ma L., Gilani A., Yi Q., Tang L. (2022). MicroRNAs as Mediators of Adipose Thermogenesis and Potential Therapeutic Targets for Obesity. Biology.

[63] Alsaggar M., Mills M., Liu D. (2017). Interferon beta overexpression attenuates adipose tissue inflammation and high-fat diet-induced obesity and maintains glucose homeostasis. Gene Ther..

[64] Wieser V., Adolph T.E., Grander C., Grabherr F., Enrich B., Moser P., Moschen A.R., Kaser S., Tilg H. (2018). Adipose type I interferon signalling protects against metabolic dysfunction. Gut.

[65] Radványi Á., Röszer T. (2024). Interleukin-6: An Under-Appreciated Inducer of Thermogenic Adipocyte Differentiation. Int. J. Mol. Sci..

[66] Woo J.G., Zhang N., Fenchel M., Jacobs D.R. (2020). Prediction of adult class II/III obesity from childhood BMI: The i3C consortium. Int. J. Obes..

[67] Ward Z.J., Long M.W., Resch S.C., Giles C.M., Cradock A.L., Gortmaker S.L. (2017). Simulation of Growth Trajectories of Childhood Obesity into Adulthood. N. Engl. J. Med..

[68] Hillier T.A., Pedula K.L., Schmidt M.M., Mullen J.A., Charles M.A., Pettitt D.J. (2007). Childhood obesity and metabolic imprinting: The ongoing effects of maternal hyperglycemia. Diabetes Care.

[69] Lukaszewski M.A., Eberle D., Vieau D., Breton C. (2013). Nutritional manipulations in the perinatal period program adipose tissue in offspring. Am. J. Physiol. Endocrinol. Metab..

[70] Sacco M.R., de Castro N.P., Euclydes V.L.V., Souza J.M., Rondó P.H.C. (2013). Birth weight, rapid weight gain in infancy and markers of overweight and obesity in childhood. Eur. J. Clin. Nutr..

[71] Vasylyeva T.L., Barche A., Chennasamudram S.P., Sheehan C., Singh R., Okogbo M.E. (2013). Obesity in prematurely born children and adolescents: Follow up in pediatric clinic. Nutr. J..

[72] Reichetzeder C. (2021). Overweight and obesity in pregnancy: Their impact on epigenetics. Eur. J. Clin. Nutr..

[73] Houde A.A., Hivert M.F., Bouchard L. (2013). Fetal epigenetic programming of adipokines. Adipocyte.

[74] Lecoutre S., Petrus P., Rydén M., Breton C. (2018). Transgenerational Epigenetic Mechanisms in Adipose Tissue Development. Trends Endocrinol. Metab..

[75] He Y., Lawlor N.T., Newburg D.S. (2016). Human Milk Components Modulate Toll-Like Receptor–Mediated Inflammation. Adv. Nutr..

[76] Newburg D.S., Walker W.A. (2007). Protection of the neonate by the innate immune system of developing gut and of human milk. Pediatr. Res..

