# Supplementary material for: Adipose Tissue Macrophages of the Human Fetus
Source: Cells. 2024 Oct 28;13(21):1787. doi: 10.3390/cells13211787 (PMC11545370; doi:10.3390/cells13211787)
Supplement: Supplementary file 1 [file cells-13-01787-s001.zip › cells-3248841-supplementary.pdf]

cells

Supplementary Materials

Adipose Tissue Macrophages of the Human Fetus

Ádám Radványi [1], Katalin Gyurina [1], Emese Rácz [2], Ilona Kovács [2], Gábor Méhes [2] and Tamás Röszer [1,*]

[1] Department of Pediatrics, Faculty of Medicine, University of Debrecen, 4032 Debrecen, Hungary; radvanyi.adam@med.unideb.hu (Á.R.); gyurina.katalin@med.unideb.hu (K.G.)
[2] Department of Pathology, Faculty of Medicine, University of Debrecen, 4032 Debrecen, Hungary; dr.racz.emese@med.unideb.hu (E.R.); dr.kovacs.ilona@med.unideb.hu (I.K.); gabor.mehes@med.unideb.hu (G.M.)
* Correspondence: roszer.tamas@med.unideb.hu

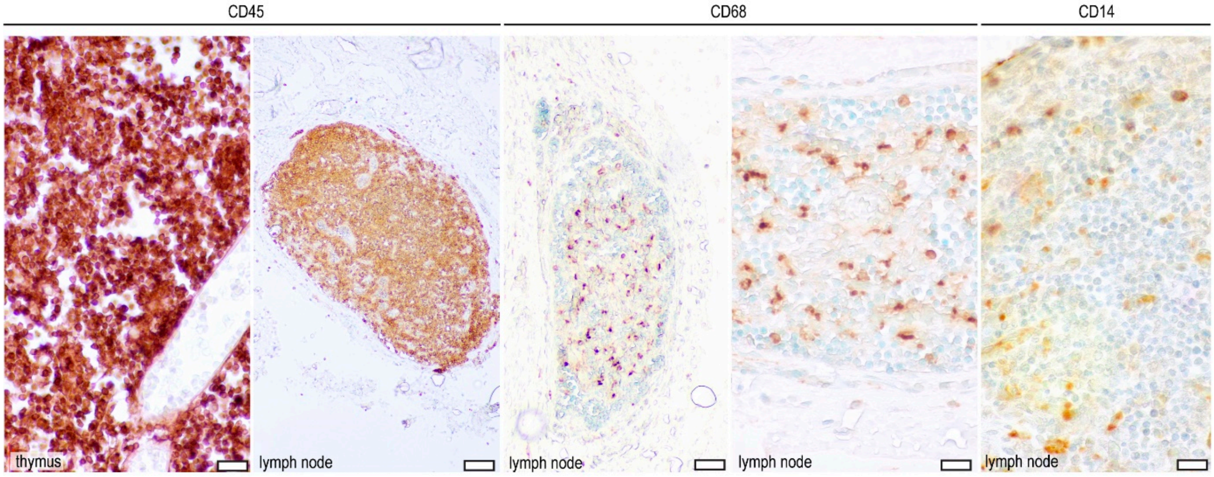

Figure S1. Macrophage labeling in fetal thymus and lymph node. Scale bars 50, 100, 100, 30, 30 μm.

Supplemental Table S1.

gender	gestational age	cause of fatality/abortion
male	17 weeks	trisomy 21
non-defined	17 weeks	trisomy 21
male	19 weeks	phocomelia
female	19 weeks	spina bifida
male	19 weeks	trisomy 21
male	20 weeks	trisomy 21
male	20 weeks	cleft lip and palate
female	20 weeks	heart defect
male	20 weeks	trisomy 21
male	21 weeks	cerebral ventriculomegaly
female	22 weeks	placental abruption
male	30 weeks	placental abruption
male	34 weeks	intrauterine asphyxia
female	36 weeks	placental abruption
male	38 weeks	placental abruption
